# A Call for an International Dental Congress

**Published:** 1890-03

**Authors:** 


### A CALL FOR AN INTERNATIONAL DENTAL CONGRESS.

On another page may be found a circular issued by the New Jersey State Dental Society, for a meeting of delegates from the different dental societies of the United States, looking to the issuing of a call for an international dental congress, to be held in 1892. When the circular was sent out, the location of the World's Fair was still in doubt, and the doubt seemed to be in favor of New York; but now that the Queen City of the West has captured the prize, it is barely possible that the New Jersey State Dental Society may not be so anxious to inaugurate the movement as it was.

The *Archives* has expressed itself as opposed to the calling of a meeting as proposed by the circular, and so must we. If such a meeting is called and a movement started to establish a congress, it will not represent the profession in America, but a small faction in the East, and will not meet with hearty support. Any movement that looks towards taking the control of the World's Fair meeting out of the hands of the American Dental Association will meet with sharp opposition. There is a fixed sentiment in the profession that any credit or benefit to be obtained from such a meeting should accrue to our already existing associations. The leading journals have expressed themselves in that way, and any attempt to use the occasion or the meeting for individual ends will meet with strong opposition from this journal. Let us throw aside all politics in the matter and make the meeting one of the grandest in the history of the American Dental Association, one noticeable for its high scientific value.

Let us not have a repetition of the difficulties which arose in the organization of the Ninth International Medical Congress, when a small faction tried to take the control of the congress out of the hands of the American Medical Association. The American Dental Association bears the same relation to the present movement that the American Medical Association did to the congress. All such movements should be under the control of delegate bodies in order to insure success, and that is the reason we have so strongly favored placing the whole matter in the hands of the American Dental Association.

